# AGE/RAGE-Induced EMP Release via the NOX-Derived ROS Pathway

**DOI:** 10.1155/2018/6823058

**Published:** 2018-03-18

**Authors:** Ying-Hua Chen, Zhang-Wei Chen, Hong-Mei Li, Xin-Feng Yan, Bo Feng

**Affiliations:** ^1^Department of Endocrinology, East Hospital, Tongji University, Shanghai 200120, China; ^2^Department of Cardiology, Shanghai Institute of Cardiovascular Diseases, Zhongshan Hospital, Fudan University, Shanghai 200032, China

## Abstract

**Objective:**

Diabetes is associated with accelerated formation of advanced glycation end products (AGEs) that are extensively found in circulating endothelial microparticles (EMPs). This study aimed to investigate whether AGEs have a direct effect on EMP formation and the possible underlying mechanism.

**Methods:**

In vitro, cultured human umbilical vein endothelial cells (HUVECs) were incubated with AGEs (200 and 400 *μ*g/ml) for 24 hours with or without pretreatment with anti-RAGE antibody, NOX inhibitor, or ROS scavenger. The number of CD31-positive EMPs was assessed by flow cytometry.

**Results:**

The number of EMPs was significantly increased in HUVECs stimulated by AGEs in a dose-dependent manner. In addition, receptors for AGEs (RAGE), NAD(P)H oxidase (NOX), and reactive oxygen species (ROS) were increased by AGEs as compared to the control group. These changes could be reversed when HUVECs were pretreated with anti-RAGE antibody. Moreover, inhibition of NOX as well as antioxidant treatment reduced the release of EMPs induced by AGEs.

**Conclusion:**

Our study suggested that AGEs increased EMP generation, which was mediated by RAGE signaling through NOX-derived ROS.

## 1. Introduction

Atherosclerosis remains the major cause of death and disability in diabetes mellitus patients due to greater severity and more diffuse distribution than in individuals without diabetes [1]. Endothelial injury is thought to be the basic pathological alteration in atherosclerosis. While multiple risk factors are associated with endothelial injury, including hyperglycemia, hyperlipidemia, and hypertension [2], growing evidence shows that endothelial microparticles (EMPs) also play an important role in the development of endothelial injury [3]. Elevated EMPs are found in most cardiovascular diseases, such as coronary heart disease, metabolic syndrome, and hypertension [4–6]. Our previous studies showed that EMP levels were elevated in type 2 diabetes and had a positive relationship with arterial function [7, 8]. Furthermore, *in vivo* and *in vitro*, isolated EMPs could impair angiogenesis, promote oxidative stress, and impair vasorelaxation [3, 9]. EMPs are not only biomarkers but also bioactive effectors of endothelial damage. Thus, it is important to fully elucidate the mechanism of EMP release in diabetes to prevent the development of vascular complications. However, the exact mechanism of EMP release in diabetes remains unclear.

Chronic hyperglycemia is associated with accelerated formation of AGEs and their interaction with the receptor (RAGE) [10–12]. The interaction between AGEs and RAGE results in the initiation of numerous signaling pathways, such as the NAD(P)H oxidase (NOX) pathway, which ultimately leads to the generation of reactive oxygen species (ROS) [13]. ROS generated by the NOX family act as second messengers regulating endothelial cell injury and contributing to the chronic complications in diabetes [14–16]. However, whether AGEs participate in EMP release remains unclear. Our previous studies had shown that EMP levels were elevated in type 2 diabetes and were positively related to HbA1c, one type of AGEs. So, we supposed that there may be a close relationship between EMP and AGEs [7]. Therefore, we designed this study to investigate whether AGEs have a direct effect on EMP formation and the possible underlying mechanism.

## 2. Methods

### 2.1. Preparation of Advanced Glycation End Products (AGEs)

Bovine serum albumin (BSA) and D-glucose (Sigma, USA) were dissolved in PBS (pH 7.2–7.4), at final concentrations of 5 g/l BSA and 50 mmol/l D-glucose. EDTA was used to reduce oxidation. Penicillin (100 U/l) and streptomycin (100 *μ*g/ml) (Sigma, USA) were added to the reaction mixture to prevent bacterial contamination. The reaction mixture was filtered through 0.22 *μ*m filter and then subjected to electrothermal incubation at 37°C for 12 weeks. At the end of the incubation period, the reaction mixture was dialyzed against sterilized PBS (pH 7.2–7.4) to remove the unconjugated glucose. The glucose in the dialyzate was <0.03 mmol/l. The reaction mixture was tested in a fluorospectrophotometer at an excitation wavelength of 370 nm, and the maximum absorption peak was measured at 440 nm to ensure that the mixture was AGEs. Then, the AGEs were freeze dried and stored at 4°C.

### 2.2. Cell Culture

Human umbilical vein endothelial cells (HUVECs) were isolated from the healthy umbilical vein with trypsin/EDTA (Sigma, USA). Cells were grown and maintained in endothelial basal medium-2 (EBM-2) (Sigma, USA) supplemented with 2% fetal bovine serum and growth factors (Sigma, USA) at 37°C in an atmosphere of 5% CO_2_. HUVECs at passage 3-4 were used for experiments. In order to investigate the effect of AGEs on the EMP releasing, we treated HUVECs with different concentrations of AGEs (200 *μ*g/ml and 400 *μ*g/ml) or BSA (400 *μ*g/ml) as the control group for 24 h. To identify the signaling pathway responsible for the AGE-mediated EMP release, we pretreated HUVECs with inhibitors (anti-RAGE antibody, NOX-1/NOX-4 inhibitor, and ROS scavenger *N*-acetylcysteine) 6 h before adding high doses of AGEs (400 *μ*g/ml, 24 h). The HUVECs were randomly assigned to six groups as follows: (1) low doses of AGEs (200 *μ*g/ml), (2) high doses of AGEs (400 *μ*g/ml), (3) pretreated with anti-RAGE antibody (5 *μ*g/ml) (ab89911, Abcam, USA) + high doses of AGEs (400 *μ*g/ml), (4) pretreated with the NOX-1/NOX-4 inhibitor (GKT137831, 10 *μ*mol/l) (S7171, Selleck Chemicals, USA) + high doses of AGEs (400 *μ*g/ml), (5) pretreated with ROS scavenger *N*-acetylcysteine (NAC, 10 *μ*mol/l) (ab143032, Abcam, USA) + high doses of AGEs (400 *μ*g/ml), and (6) the control group, 400 *μ*g/ml BSA.

### 2.3. Flow Cytometric Detection of Endothelial Microparticles (EMPs)

**Figure 1:**
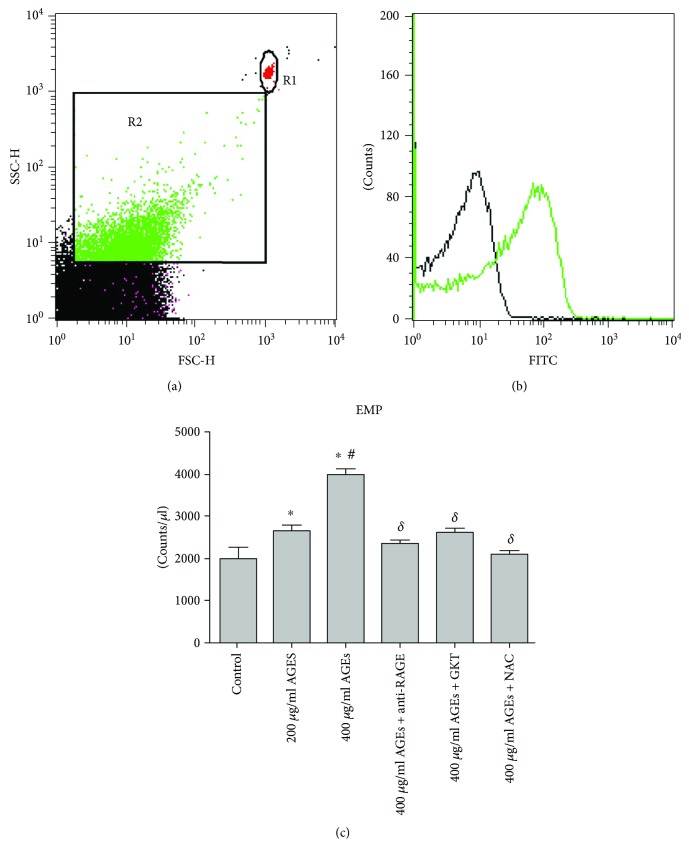
Flow cytometry detection of endothelial microparticles (EMPs). (a) EMPs (R2) and calibrator beads (R1) are represented on a forward scatter/side scatter dot plot histogram. (b) EMPs were analyzed for fluorescence associated with CD31-FITC. (c) Levels of EMP in HUVECs. The level of EMPs upon AGE stimulation was greatly enhanced. Pretreatment with anti-RAGE antibody (5 *μ*g/ml), NOX-1/NOX-4 inhibitor (GKT137831, 10 *μ*mol/l), or ROS scavenger *N*-acetylcysteine (NAC, 10 *μ*mol/l) suppressed high doses of AGE-induce EMP releasing (^∗^*p* < 0.05 versus control, ^#^*p* < 0.05 versus 200 *μ*g/ml AGEs, and ^δ^*p* < 0.05 versus 400 *μ*g/ml AGEs). Data are expressed as mean ± SD in each group.

Culture supernatants were collected and cleared from cell fragments by centrifugation at 4300*g* for 5 min. The supernatant was then ultracentrifuged at 200,000 rpm for 120 min at 10°C. Pelleted EMPs were resuspended in PBS (pH 7.2–7.4) and immediately used. 50 *μ*l EMP suspension was incubated with 3 *μ*l of anti-human CD31-FITC (557508, BD, USA) or 3 *μ*l of isotypic immunoglobulins IgG1-FITC (551954, BD, USA). The samples were then incubated for 20 min at room temperature in the dark with gentle shaking. After labeling, the samples were analyzed by flow cytometry (FACS Calibur, BD, USA) [7]. For flow cytometry assay, light scatter and fluorescence channels were set at log gain. EMP gate (R2, Figure 1(a)) was defined by excluding the first forward scatter channel that contained most of the background noise and by including an internal standard bead (0.8 *μ*m, LB8, Sigma, USA). Only events included within this gate were further analyzed for fluorescence. For EMP numeration, calibrator beads (3 *μ*m, LB30, Sigma, USA) were added to the sample. EMP was defined as CD31-positive particles (Figures 1(a) and 1(b)).

### 2.4. RNA Isolation and Quantitative RT-PCR

Total RNA was extracted using the TRIzol reagent (Invitrogen, CA) according to the manufacturer's protocol. Reverse transcription was performed using the cDNA reverse transcription kit (Applied Biosystems), and subsequent real-time RT-PCR analyses were performed using SYBR Green (Takara, JAP). The relative levels of transcripts were calculated using the 2^−ΔΔCt^ method after normalizing with GAPDH as the internal control. Primers used are shown in Table 1.

### 2.5. Protein Isolation and Western Blot Analysis

Cell extracts were prepared with a lysis buffer according to the manufacturer's instructions. Forty-microgram proteins were separated in 10% SDS-PAGE and transferred to nitrocellulose membranes. The membranes were probed with NOX-1 (ab55831, Abcam, USA), NOX-2 (ab80508, Abcam, USA), NOX-4 (ab154244, Abcam, USA), RAGE (ab3611, Abcam, USA), or GAPDH (ab8245, Abcam, USA) antibodies at 4°C overnight and subsequently with peroxidase-conjugated secondary antibody for 1 h at room temperature. Proteins were visualised using electrochemiluminescent reagents.

### 2.6. Evaluation of ROS Production by Flow Cytometry

ROS levels were detected using a flow cytometer and a microplate spectrophotometer (Molecular Devices, USA). Cells were harvested and washed with PBS and suspended in 10 *μ*M 5(6)-carboxy-2′,7′-dichlorodihydrofluorescein diacetate (carboxy-H2DCFDA; no: 88-5930-74, Invitrogen) at 37°C for 20 min. The cells were then washed twice with PBS and subjected to flow cytometry analysis.

### 2.7. Statistical Analysis

Continuous variables, presented as mean ± SD, were analyzed for distribution status, and continuous variables with normal distribution were compared using Student's *t*-test for analysis; nonnormal distribution data were tested with two-tailed Mann–Whitney *U* test. Comparisons between multiple groups were performed using one-way ANOVA. All data were analyzed with SPSS software (version 19.0). All *p* values were two sided, and *p* < 0.05 was considered statistically significant.

## 3. Results

### 3.1. AGEs Increased EMP Release

The effect of AGEs on EMP formation in HUVECs was examined *in vitro*. Cells were treated with or without 200 *μ*g/ml AGEs, 400 *μ*g/ml AGEs, or control for 24 hours. Treatment with 200 *μ*g/ml AGEs led to a significant increase in the level of EMPs as compared to the control group (2650 ± 306 counts/*μ*l versus 1999 ± 579 counts/*μ*l, *p* < 0.05). Treatment with 400 *μ*g/ml AGEs showed a dramatic increase in EMPs as compared to the 200 *μ*g/ml AGE group (3979 ± 376 counts/*μ*l versus 2650 ± 306 counts/*μ*l, *p* < 0.05). AGEs significantly increased the release of EMPs in a dose-dependent manner (Figure 1(c)).

### 3.2. AGE-RAGE Interaction Participated in the Release of EMP

**Figure 2:**
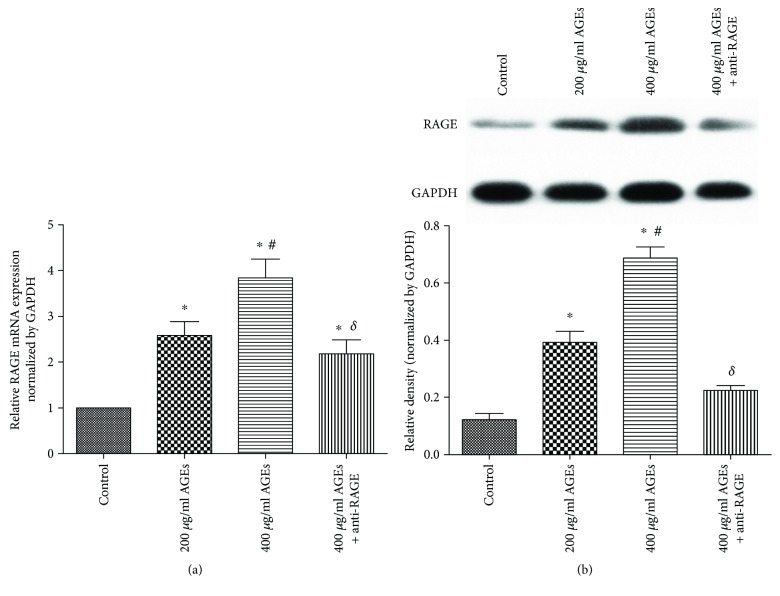
Effect of AGEs on RAGE expression in HUVECs. (a) The mRNA expression of RAGE was determined using quantitative real-time PCR. (b) Protein expression of RAGE in each group was analysed by Western blot. Data are expressed as mean ± SD in each group. ^∗^*p* < 0.05 versus control, ^#^*p* < 0.05 versus 200 *μ*g/ml AGEs, and ^δ^*p* < 0.05 versus 400 *μ*g/ml AGEs.

AGE-RAGE interactions play an important role in many pathologies, but whether they participate in AGE-induced EMP release remains unclear. Our investigation showed that AGEs could induce a rapid increase in RAGE mRNA and protein (Figure 2). Pretreatment of cells with anti-RAGE antibody for 6 h and stimulation of cells with high doses of AGEs (400 *μ*g/ml) showed that the levels of EMPs were dramatically decreased (2364 ± 170 counts/*μ*l versus 3979 ± 376 counts/*μ*l, *p* < 0.05) (Figure 1(c)) and RAGE mRNA and protein expression was suppressed (Figure 2) as compared to the 400 *μ*g/ml AGE group. These findings suggested that activation of RAGE was involved in AGE-induced EMP release.

### 3.3. The NOX/ROS Pathway Participated in AGE/RAGE-Induced EMP Release

**Figure 3:**
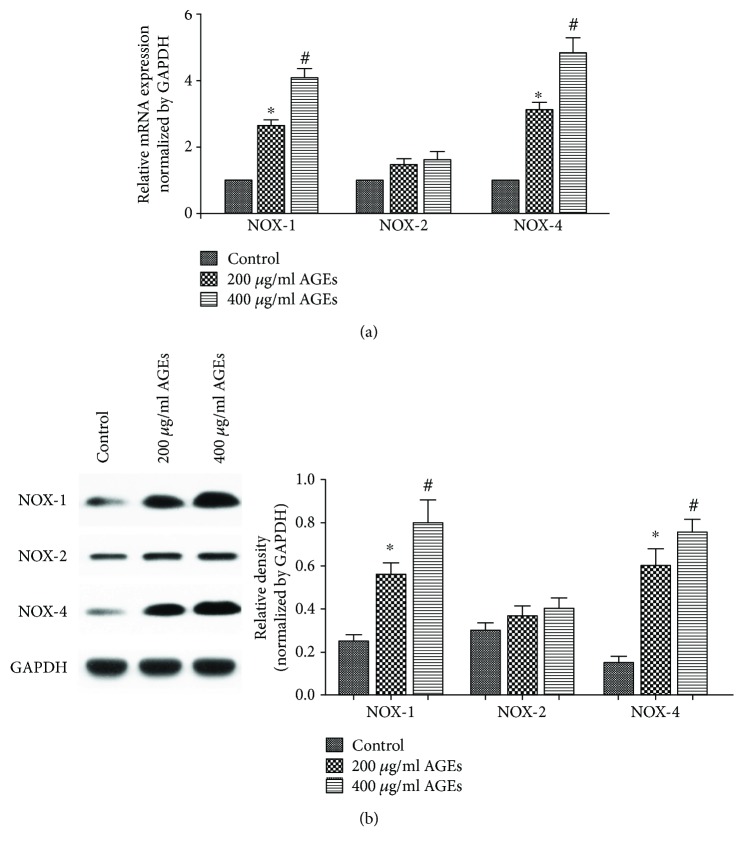
Effect of AGEs on NOX expression in HUVEC. (a) The mRNA expression of NOX-1, NOX-2, and NOX-4 was determined using quantitative real-time PCR. (b) Protein expression of NOX-1, NOX-2, and NOX-4 in each group was analysed by Western blot. Data are expressed as mean ± SD in each group. ^∗^*p* < 0.05 versus control; ^#^*p* < 0.05 versus 200 *μ*g/ml AGEs.

**Figure 4:**
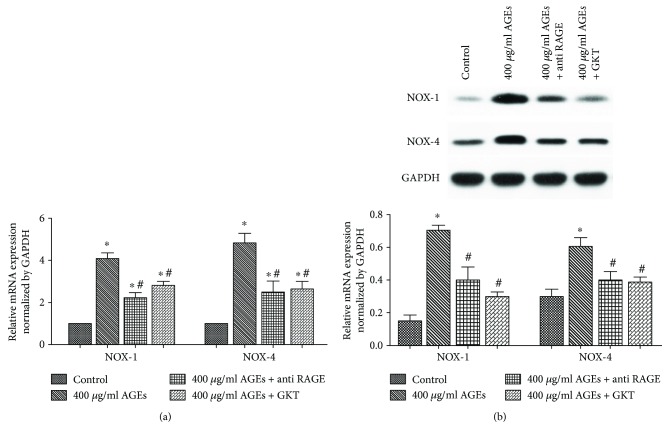
NOX expression in HUVEC suppressed by anti-RAGE antibody and NOX inhibitor. (a) The mRNA expression of NOX-1 and NOX-4 was determined using quantitative real-time PCR. (b) Protein expression of NOX-1 and NOX-4 in each group were analysed by Western blot. Data are expressed as mean ± SD in each group. ^∗^*p* < 0.05 versus control; ^#^*p* < 0.05 versus 400 *μ*g/ml AGEs.

The interaction of AGEs and RAGE results in the initiation of several signaling pathways. To examine whether oxidative stress, as a downstream pathway, was responsible for the AGE/RAGE-induced EMP release, we examined the roles of NOX and ROS. As shown in Figure 3, AGEs increased not only the formation of EMPs but also the mRNA and protein expression of NOX-1 and NOX-4, with no change in the expression of NOX-2 (Figure 3). However, pretreatment of cells with anti-RAGE antibody suppressed the expression of NOX-1 and NOX-4 (Figure 4). Then, we pretreated HUVECs with the NOX-1/NOX-4 inhibitor (GKT 137831, 10 *μ*mol/l) for 6 h before adding 400 *μ*g/ml AGEs and found a significant reduction in EMPs as compared to HUVECs grown in 400 *μ*g/ml AGEs alone (2619 ± 227 counts/*μ*l versus 3979 ± 376 counts/*μ*l, *p* < 0.05) (Figure 1(c)).

AGEs significantly increased ROS production in a dose-dependent manner, while anti-RAGE, NOX-1/NOX-4 inhibitor, or ROS scavenger *N*-acetylcysteine could reverse these effects (Table 2). Meanwhile, pretreatment of HUVECs with ROS scavenger *N*-acetylcysteine (NAC, 10 *μ*mol/l) for 6 h followed by stimulation with high doses of AGEs (400 *μ*g/ml) led to a significant decrease in the level of EMPs (2113 ± 154 counts/*μ*l versus 3979 ± 376 counts/*μ*l, *p* < 0.05) as compared to HUVECs grown in high doses of AGEs alone (Figure 1(c)). These findings suggested that NOX-derived ROS was involved as a downstream factor in AGE/RAGE-induced EMP release.

## 4. Discussion

The present study showed that AGEs significantly promoted EMP generation *in vitro* in a dose-dependent manner. In addition, AGEs increased the expression of RAGE, NOX, and ROS. Pretreatment of HUVECs with anti-RAGE antibody could reverse this effect. Furthermore, when pretreated with the NOX inhibitor or ROS scavenger, the release of EMP and ROS induced by AGEs was inhibited. These findings suggested that AGEs increase EMP generation, an effect mediated by RAGE that signals through NOX-derived ROS.

EMPs, vesicles released from the plasma membrane surface of endothelial cells, are augmented in most cardiovascular diseases when comparing a patient population with a matched group of healthy subjects [3–8]. An elevation of EMP levels is considered as a biomarker of vascular endothelial damage [17]. Furthermore, EMPs show pathogenicity in impairing angiogenesis, promoting oxidative stress, and impairing vasorelaxation [3, 9]. Many factors have been implicated as inducers of EMP release, such as CRP, ATII, and uremic toxins [18–20]. However, the exact mechanism of EMP release in diabetes remains unclear.

Our previous studies had shown that EMP levels were elevated in type 2 diabetes and were positively related to HbA1c, a glycated protein [7]. Diabetes is associated with the formation of AGEs, a heterogeneous group of molecules resulting from the nonenzymatic glycation of proteins, lipids, and nucleic acids. AGE levels were correlated with diabetic complications including diabetic retinopathy, nephropathy, and cardiovascular disease [21, 22]. High levels of AGEs were associated with atherosclerosis plague progression [23]. The importance of AGEs in diabetes was proven using inhibitors of advanced glycation to retard the development of vascular disease without directly affecting the level of glucose [24]. Furthermore, food with high levels of AGEs was shown to accelerate atherosclerosis without affecting glycemic control [25]. Despite the important contribution of AGEs to accelerated atherosclerosis, whether AGEs could influence EMP formation remained unclear. In order to investigate whether AGEs directly influence EMP formation, we exposed HUVECs to different doses of AGEs, measured EMPs by flow cytometry, and found that AGEs directly stimulated HUVECs to form EMPs in a dose-dependent manner. Furthermore, AGEs increased the levels of EMPs and RAGE. When exposed to anti-RAGE antibody, AGE-induced EMP generation was inhibited, indicating the role of AGEs/RAGE in EMP release.

Indeed, the activation of RAGE by AGEs results in the initiation of various signal transduction cascades, including NADPH oxidases, mitogen-activated protein kinases, p21 ras, ERK, p38, and protein kinase C [13]. There is increasing evidence that ROS induced by NADPH oxidases not only causes cell damage but also acts as second messengers implicated in proliferation, differentiation, and apoptosis, which finally leads to accelerated atherosclerosis in diabetes [26]. Diabetes is accompanied by increased generation of ROS, particularly in patients with complications [16]. Multiple pathways may lead to ROS production. However, the NOX family is a major source of ROS in endothelial cells [26]. The NOX family comprises of seven members: NOX-1, NOX-2, NOX-3, NOX-4, NOX-5, Duox1, and Duox2 [26]. Four NOX proteins (NOX-1, NOX-2, NOX-4, and NOX-5) are expressed in endothelial cells [26]. NOX-1, NOX-2, and NOX-4 activities were shown to contribute to vascular pathology [27–29]. However, the role of NOX-5 in vascular pathology is difficult to assess since this enzyme is found in the human vasculature, but not in mice and rats [26].

In order to identify the signaling pathway responsible for the AGEs/RAGE-mediated EMP release, we examined the role of NOX and ROS and found that AGEs/RAGE increased the levels of EMPs, NOX, and ROS, while the NOX inhibitor as well as ROS scavenger reversed these effects. These results demonstrated that NOX-derived ROS participated in AGE/RAGE-induced EMP release. Additionally, both NOX-1 and NOX-4 participated in the process of AGE-induced EMP release. Although NOX-2 was previously shown to play an important role in endothelial vascular pathobiology [28], we did not observe any effect of NOX-2 in AGE-induced EMP release. GKT137831, a member of the pyrazolopyridine dione family, is a specific inhibitor of both NOX-1 and NOX-4. In this study, GKT137831 inhibited AGE-induced EMP generation, indicating that the NOX-1/NOX-4 inhibitor could serve as a novel therapeutic target in AGE-related vascular endothelial injury. Indeed, NOX-derived ROS was associated with atherosclerotic plaques in diabetic mice, and blockade of NOX-derived ROS using GKT137831 prevented the diabetes-mediated increase in the atherosclerotic plaque area and also significantly reduced vascular ROS [30].

## 5. Conclusions

In conclusion, our data showed that AGEs/RAGE induced EMP release via the NOX-derived ROS pathway, which provides new insights into the molecular mechanisms of EMP release in diabetes.

## Figures and Tables

**Table 1:** The sequences of the sense primers and antisense primers used in this study.

mRNA	Sense primer	Antisense primer
RAGE	5′-GAAACTGAACACAGGCCGGA-3′	5′-CACGGACTCGGTAGTTGGAC-3′
NOX-1	5′-CCGCACACTGAGAAAGCAAT-3′	5′-CCGGACAATTCCACCAAT-3′
NOX-2	5′-CAGCCTGCCTGAATTTCAACT-3′	5′-GGAGAGGAGATTCCGACACACT-3′
NOX-4	5′-TGTTGGGCCTAGGATTGTGTT-3′	5′-AGGGACCTTCTGTGATCCTCG-3′
GAPDH	5′-TCATCAGCAATGCCTCCTGTACCA-3′	5′-TATTTGGCAGGTTTCTCCAGACGG-3′

**Table 2:** Level of ROS (mean fluorescence intensity units) in HUVEC.

Control	200 *μ*g/ml AGEs	400 *μ*g/ml AGEs	400 *μ*g/ml AGEs + anti-RAGE	400 *μ*g/ml AGEs + GKT	400 *μ*g/ml xAGEs + NAC
23 ± 4	132 ± 32^∗^	233 ± 17^∗^^#^	176 ± 24^δ^	194 ± 31^δ^	144 ± 23^δ^

^∗^ *p* < 0.05 versus control; ^#^*p* < 0.05 versus 200 *μ*g/ml AGEs; ^*δ*^*p* < 0.05 versus 400 *μ*g/ml AGEs.
